# Automatic Identification of Subtechniques in Skating-Style Roller Skiing Using Inertial Sensors

**DOI:** 10.3390/s16040473

**Published:** 2016-04-02

**Authors:** Yoshihisa Sakurai, Zenya Fujita, Yusuke Ishige

**Affiliations:** 1 Department of Sports Science, Japan Institute of Sports Sciences, 3-15-1 Nishigeoka, Kita-ku, Tokyo 115-0056, Japan; yusuke.ishige@jpnsport.go.jp; 2 Faculty of Sport Sciences, Waseda University, 2-579-15 Mikajima, Tokorozawa, Saitama 359-1192, Japan; zenya.fujita@aoni.waseda.jp

**Keywords:** cross-country skiing, accelerometer, gyroscope

## Abstract

This study aims to develop and validate an automated system for identifying skating-style cross-country subtechniques using inertial sensors. In the first experiment, the performance of a male cross-country skier was used to develop an automated identification system. In the second, eight male and seven female college cross-country skiers participated to validate the developed identification system. Each subject wore inertial sensors on both wrists and both roller skis, and a small video camera on a backpack. All subjects skied through a 3450 m roller ski course using a skating style at their maximum speed. The adopted subtechniques were identified by the automated method based on the data obtained from the sensors, as well as by visual observations from a video recording of the same ski run. The system correctly identified 6418 subtechniques from a total of 6768 cycles, which indicates an accuracy of 94.8%. The precisions of the automatic system for identifying the V1R, V1L, V2R, V2L, V2AR, and V2AL subtechniques were 87.6%, 87.0%, 97.5%, 97.8%, 92.1%, and 92.0%, respectively. Most incorrect identification cases occurred during a subtechnique identification that included a transition and turn event. Identification accuracy can be improved by separately identifying transition and turn events. This system could be used to evaluate each skier’s subtechniques in course conditions.

## 1. Introduction

In skating-style cross-country skiing, based on the course terrain and their skiing velocity, skiers mainly use three subtechniques [1]. In the skating phase, skiers glide on one ski from the push-off movement of the other leg with a V-shaped ski orientation. In the pushing phase, skiers extend their upper extremities and push both poles backward in order to produce the propulsive force. In these subtechniques, skiers perform skating and pushing movements using different timings. The V1 skating technique (V1) is generally considered to be a steep uphill technique and uses an asymmetrical pole push with every second leg push-off. The V2 skating technique (V2) is mainly used on level terrain up to moderate uphill inclines, and is performed with a symmetrical double poling action for each skating push-off. The V2-alternate skating technique (V2A) is used on level terrain with a symmetrical double poling action with every second leg push-off. V1 and V2A have the same number of pole movements and leg push-offs. However, these two movements occur with different timings in the two subtechniques.

Some studies have reported the differences of each subtechnique based on physiology and biomechanics. It was found that there is no difference in the heart rate and oxygen cost among subtechniques on flat terrain [2]. Bilodeau *et al.* [3] compared the skiing velocities and physiological responses of these three subtechniques over a flat, an uphill, and a downhill section, as well as a complete course. The results showed that there is no significant difference in the skiing velocity, estimated oxygen uptake, and average heart rate among these three subtechniques. Similarly, there is no difference among the three subtechniques in the skiing velocity at maximum speed on a flat terrain, although V1 exhibits a higher cycle rate than V2 and V2A. Furthermore, V2 has a longer cycle length than V1 and V2A [1]. In contrast, it has been reported that with an increase in inclination, V2 increasingly requires a higher oxygen cost compared to V1 [4]. In addition, it has been reported that V1 is faster than V2 on a 5.0° uphill grade and that the average total cycle force of poles and skis in V1 is higher than that in V2 on a 7–10° uphill grade [5]. These data suggest that there are differences in the oxygen cost and exerted force on an uphill terrain. Furthermore, V2A is slower than the other two subtechniques on a 5.0°uphill grade when skating at the same intensity [6]. Hence, top-level skiers strategically choose different subtechniques to obtain higher speed and efficiency. Andersson *et al.* [7] examined subtechnique selection during a simulated sprint time trial. The distributions of V1, V2, and V2A were 31%, 63%, and 6%, respectively. The results showed that sprint skiing performance is primarily related to uphill performance and better utilization of V2. Therefore, it is important to identify subtechniques during skiing to enhance skiers’ performance.

In several recent studies, small inertial sensors have been used to analyze cross-country skiing techniques. It has been shown that the subtechniques can be classified visually using the acceleration and angular velocity data from a microsensor located on the upper back [8]. It was found that a difference in hip movements between V1 and V2 can be observed using a tri-axial accelerometer placed on the sacrum [9]. These studies demonstrated the possibility of using inertial sensors to identify subtechniques. A new automatic algorithm has been developed to classify the skating style using a smartphone accelerometer attached to the chest and a machine learning technique [10,11]. However, it is difficult to calculate spatio-temporal variables such as the cycle time, poling time, and recovery time. In some studies, small inertial sensors have been mounted on poles, ski boots, roller skis, and wrists. It has been shown that the acceleration recorded by the pole accelerometer can detect pole hits and lifts, and that recorded by the heel of ski boots can detect ski lifts [12]. Fasel *et al.* [13] showed that the cycle duration, ski thrust duration, cycle speed, and cycle length of the diagonal stride can be calculated accurately using inertial sensors fixed to the pole and roller ski. Myklebust *et al.* [12] used the time of pole/ski hits and lifts to classify the subtechniques. However, this method requires many subject-specific thresholds for detecting the timing and the classification of subtechniques. Sakurai *et al.* [14] identified classical-style subtechniques automatically using inertial sensor data from both the wrists and roller skis. The subtechniques of skating-style cross-country skiing exhibit different arm and ski movement patterns and timings. Therefore, the measurements from arms and skis are considered to be particularly effective in identifying the subtechniques. Furthermore, the use of inertial sensors located on wrists and skis can be analyzed with spatio-temporal analysis for skating-style skiing. Hence, the current study aims to develop an automated subtechnique identification system using inertial sensors.

## 2. Methods

### 2.1. Development of an Automated Identification System

#### 2.1.1. Pre-Experiment

A pre-experiment was conducted to develop an automated identification system for skating-style subtechniques. A male cross-country skier (age: 22 years; height: 1.75 m; weight: 71.0 kg) participated in this study. The subject provided informed consent prior to the experiment. The subject used his own racing poles and roller skis (MS610C, Marwe Roller Skis, Hyvinkää, Finland) during the test. Four inertial sensors (LP-WS0901, 3-axis accelerometer: ±50 G; 3-axis gyroscope: ±1500 °/s, Logical Product Corp., Fukuoka, Japan) were used in this study. The data from the 3-axis accelerometer and gyroscope were synchronously written to the internal memory in each sensor. All sensors were wirelessly controlled by an application (SS-WSAP01, Logical Product Corp., Fukuoka, Japan). The sensors were worn on the back sides of both wrists of the subject using wrist pouches and were also attached to both his roller skis. The test was conducted at sub-maximal velocity using all the skating-style subtechniques with the right or left side being dominant (V1R, V1L, V2R, V2L, V2AR, and V2AL) on an asphaltic road. Angular velocities and accelerations were sampled at a rate of 100 Hz and stored by each sensor. The subject was videotaped using a digital video camera (HDR-CX700C, Sony, Tokyo, Japan) to identify the subtechniques employed.

#### 2.1.2. Definition of One Cycle

The obtained data were processed offline using MATLAB R2011a (The MathWorks, Inc., Natick, MA, USA). All raw accelerations and angular velocities obtained by the sensors were smoothed using a Butterworth low-pass digital filter with cutoff frequencies of 1 and 3 Hz. First, we defined the backswing phase of each upper extremity using the 1 Hz low-pass filtered angular velocity of the forearms corresponding to the mediolateral axis. Pole contact was defined as the local maximum point during each backswing phase—which had the largest amount of change between the local maximum and the previous local minimum of the raw forearm acceleration, whose axis was parallel to the pole at the instant of contact. When the contact point was less than 0.250 s apart from the previous one, the one that exhibited lower raw acceleration at each contact point was eliminated. The contact of both arms was defined as the contact whose difference between the right and left contacts was less than 0.025 s. The other contacts were defined as the right or left contact. The contact points were used as the start and end points of one cycle.

#### 2.1.3. Definition of Recovery Motion

**Figure 1:**
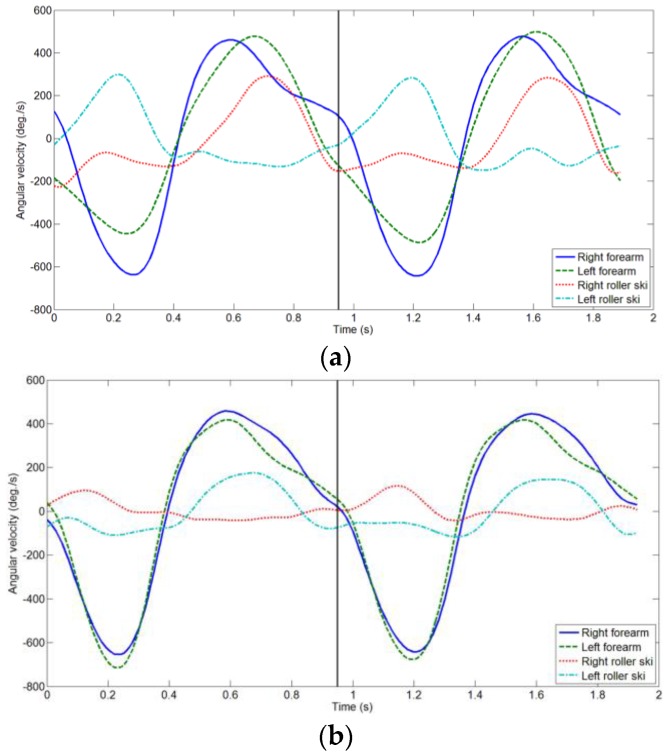
Angular velocities of forearms in the sagittal plane (forward swing/backswing) and roll angular velocities of roller skis (inward/outward) during two cycles of the V1 skating technique: (**a**) V1R; (**b**) V1L. Angular velocities were filtered with a 3 Hz low-pass filter. The vertical black line indicates the start of the second cycle.

**Figure 2:**
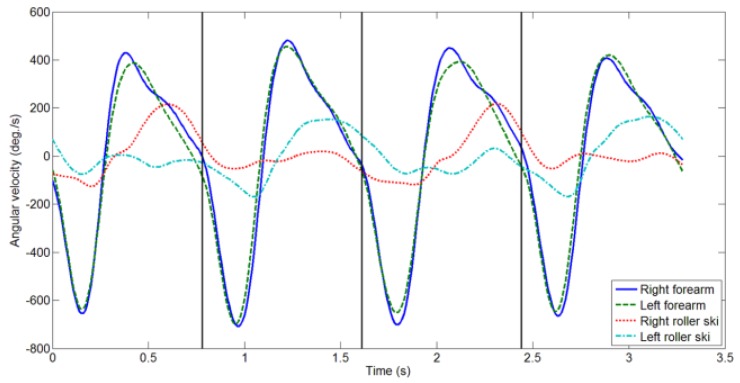
Angular velocities of forearms in the sagittal plane (forward swing/backswing) and roll angular velocities of roller skis (inward/outward) during two cycles of successive use of the V2R and V2L skating techniques. Angular velocities were filtered with a 3 Hz low-pass filter. The vertical black lines indicate the start of each cycle.

**Figure 3:**
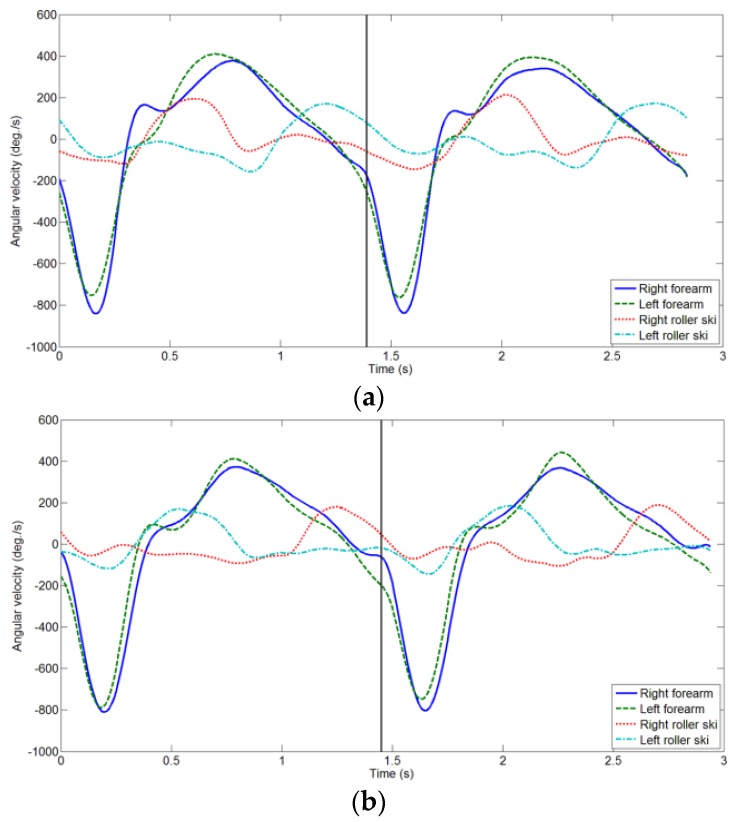
Angular velocities of forearms in the sagittal plane (forward swing/backswing) and roll angular velocities of roller skis (inward/outward) during two cycles of the use of the V2A skating technique: (**a**) V2AR; (**b**) V2AL. Angular velocities were filtered with a 3 Hz low-pass filter. The vertical black line indicates the start of the second cycle.

The roller ski shows the internal tilt for edging during the push-off movement. Subsequently, the roller ski rolls outward during the recovery phase (Figure 1, Figure 2 and Figure 3). Thus, any recovery motion was identified using the 3 Hz low-pass-filtered roll angular velocity of the roller skis. The recovery motion was defined as the roll angular velocity of the roller ski with a maximum value of over 25 × pitch °/s. This threshold was determined empirically.

#### 2.1.4. Detection of Main Subtechniques

**Figure 4:**
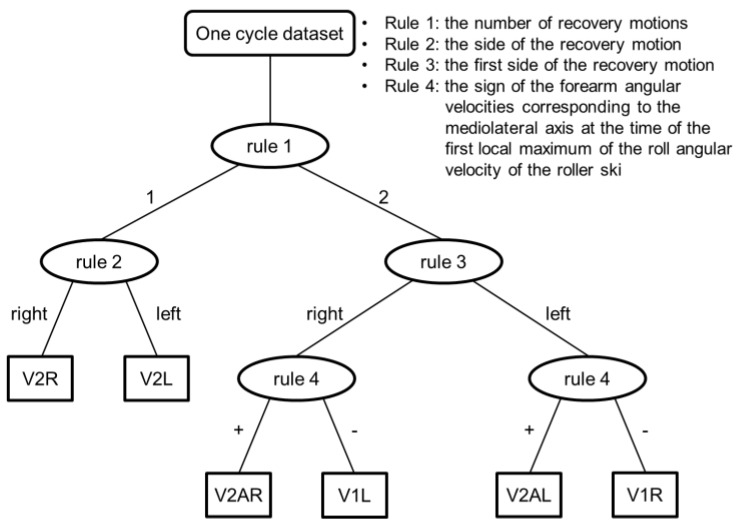
Decision tree for the classification main subtechniques.

The decision tree for the classification of main subtechniques has five decision nodes and six leaves, as illustrated in Figure 4. V2 has only one recovery motion of the right or left roller ski during the arm’s forward swing phase (Figure 2) whereas V1 and V2A have two recovery motions (Figure 1 and Figure 3). Therefore, V2 was defined by Rule 1, *i.e.*, by the number of recovery motions. V2R was defined as one recovery motion with the right roller ski and V2L with the left roller ski by Rule 2, *i.e.*, by the side of the recovery motion (Figure 4).

Figure 1 and Figure 3 show the angular velocities of the forearms and roller skis during V1 and V2A, respectively. V1 and V2A have both right and left recovery motions and similar angular velocity histories. V1R and V2AL have the recovery motions in the order of right and left (Figure 1a and Figure 3b); V1L and V2AR have recovery motions in the order of left and right (Figure 1b and Figure 3a). Therefore, V1 and V2A were divided into two groups based on the first side of the recovery motion (Rule 3 in Figure 4). After pole contact, skiers recovered the weaker side’s roller ski during the arm’s backswing motion, and there was a strong side roller ski pushing and recovery phase during the arm’s forward swing motion in V1 (Figure 1). On the other hand, the skiers recovered the roller ski after the pole push-off in V2A (Figure 3). Therefore, V1 and V2A were distinguished using the sign of the 1 Hz low-pass-filtered forearm angular velocity corresponding to the mediolateral axis at the time of the first local maximum of the roll angular velocity of the roller ski; positive and negative values are classified as V2A and V1, respectively (Rule 4 in Figure 4).

#### 2.1.5. Exceptions

There are other two subtechniques besides the above three major subtechniques in skating-style cross-country skiing. The first one is a transition from a subtechnique to another one that has a different movement pattern compared to the major subtechniques. The other one is a turn during a curve. Therefore, a new subtechnique, “V4,” was introduced to represent the transition and turn. If the identified subtechniques were a sequence needed to perform the transition (e.g., before or after V1R is the subtechnique, except for V1R) and usually did not perform it (e.g., continuous V2R or V2L), the first subtechnique of the sequence was changed to V4. Table 1 shows the exception procedure of the sequences. Row A shows the first subtechnique of the sequence, Row B shows the subtechniques that can follow the first subtechnique, and Row C shows the subtechniques that require a transition after the first subtechnique.

### 2.2. Validation Experiment

#### 2.2.1. Subjects

Eleven college cross-country skiers (seven females and four males) and four male college Nordic combined skiers belonging to the Ski Association of Japan participated in this study. The anthropometric and physical performance characteristics of the subjects are presented in Table 2. The subjects had no known disorders that would influence their skiing performance. Before the experiment, the purpose and procedures of this study were explained to each subject, and written informed consents were obtained from all of them. The experimental procedure was approved by the Ethical Committee of the Japan Institute of Sports Sciences.

#### 2.2.2. Protocol

**Figure 5:**
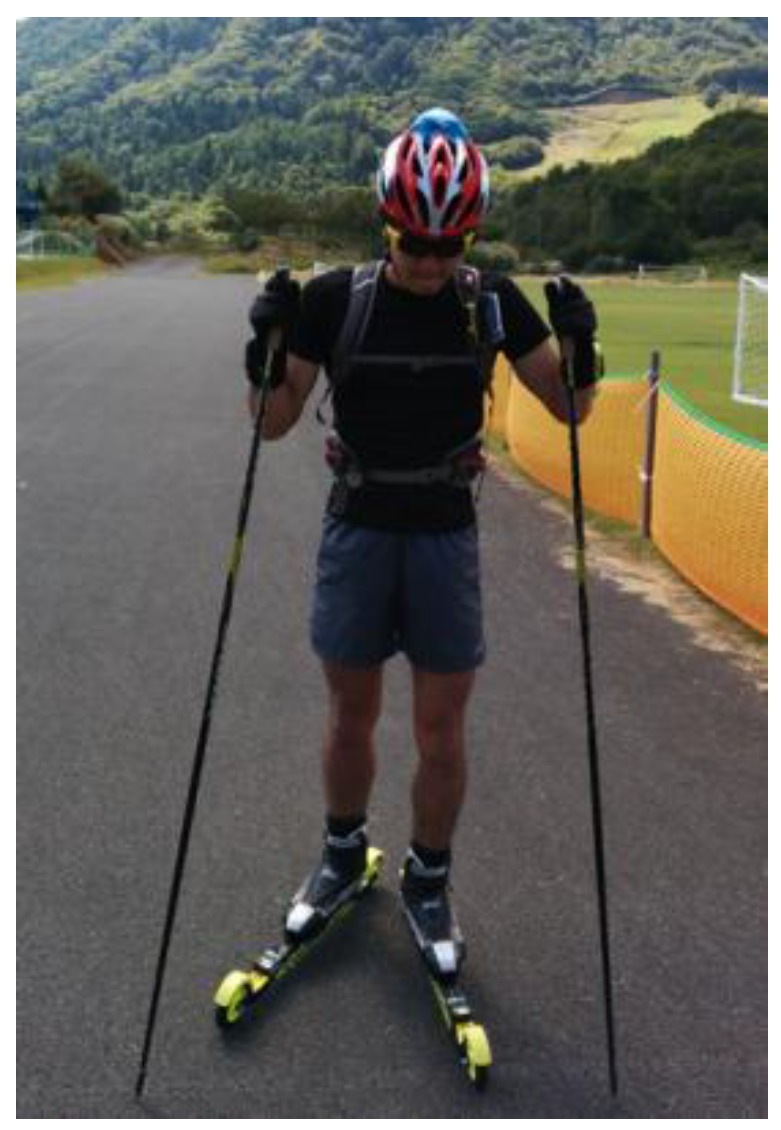
Locations of inertial sensors on wrists and in front of the bindings of roller skis. A video camera is located on the left shoulder strap.

In the experiments, all subjects used their own racing poles and racing roller skis (MS610C, Marwe Roller Skis, Hyvinkää, Finland). As in the pre-experiment, four sensors were attached: two on the wrists and two on the roller skis of the subjects. The rollers of the subjects and the movements of the pole tips were recorded using a compact digital video camera (HDR-AS15, Sony Inc., Tokyo, Japan). The camera was fixed with a downward inclination to the left shoulder strap of a tight-fitting backpack (Figure 5). All subjects skied through a 3450 m undulating roller ski course using the skating style at their maximum speed.

#### 2.2.3. Data Analysis

Subtechniques were detected using an automatic identification system that was developed based on the pre-experiment results. The actual subtechniques used were determined from the video using the movements of the poles and the roller skis. This check was carried out visually by one of the authors who was a past ski racer and is a present coach with 18 years of cross-country skiing experience. The total number of cycles and the number of cycles of each subtechnique were calculated using both the automatic and visual methods. These results were presented in the form of a confusion matrix.

## 3. Results

Table 3 shows the confusion matrix of the subtechniques obtained using the automatic and visual methods. A total of 6768 cycles (range: 385–525) subtechniques, including 127 V1R (range: 0–51), 41 V1L (range: 0–22), 1950 V2R (range: 99–200), 1955 V2L (range: 93–202), 805 V2AR (range: 0–113), 631 V2AL (range: 0–167), and 1259 V4 (range: 63–102), were identified using the visual method. A breakdown of 1259 V4 by the visual method was 66 “transition” (range: 1–9) and 1193 “turn” (range: 54–101). The subtechniques identified using the visual method were assumed to be correct and were used as a gold standard. The numbers of each subtechnique correctly identified by automatic identification were 120, 40, 1920, 1932, 767, 587, and 1047 for V1R, V1L, V2R, V2L, V2AR, V2AL, and V4, respectively. The accuracy of automatic identification for all subtechniques for all subjects was 94.7% ± 3.0% (89.0–98.3%). The identification precision for V1R, V1L, V2R, V2L, V2AR, V2AL, and V4 were 87.6%, 87.0%, 97.5%, 97.8%, 92.1%, 92.0%, and 91.7%, respectively. V4, whose recall was 83.2%, was the most incorrectly identified subtechnique.

## 4. Discussion

The automatic identification method for skating-style subtechniques correctly identified 6413 subtechnique cycles out of a total of 6768 cycles, which indicates an accuracy of 94.8%. This result implies that it is possible to identify the skating-style subtechniques used by many cross-country skiers using the proposed automatic identification method. However, there were a total of 355 incorrect identifications, 54% of which occurred during V4 identification. In addition, 27% of them identified the main subtechniques as V4. The most common incorrect identification among the main subtechniques was between V2 and V2A. There is a difference in the number of recovery motions between the two subtechniques. V2 has only one recovery motion and V2A has right and left recovery motions during one cycle. Therefore, identification accuracy can be improved by modifying the definition of the recovery motion.

In this study, V4 was defined as “transition” and “turn” because it is impossible to distinguish these events from only inertial sensor data in cross-country circuit skiing. Therefore, the automatic identification did not classify V4 directly and determined it via the exception procedure. The 5.2% and 94.8% of V4 by the visual method were “transition” and “turn”, respectively. Therefore, the correct identification of “turn” is important to improve the accuracy of this method. As for the transition, the degree of correct identification was negatively correlated to the number of transitions between subtechniques [11]. Therefore, the correct identification of “transition” will also improve the accuracy of this method. As shown in Table 2, there are 26 possible transition patterns from one subtechnique to another. Therefore, if the sensor data were available for all transitions, it may be possible to produce an identification system using signal processing or machine learning [10,11].

On the other hand, a skier generally uses a stepping action during V1, V2, or V2A to change the skiing direction during a turn. Therefore, the time histories of the inertial sensors were similar to those of the main subtechniques. Furthermore, there are many possible turning styles that can be produced by changing the step timing and number of steps. Therefore, it would be impossible to classify a turn by using sensor data only.

A global navigation satellite system (GNSS) or a global positioning system (GPS) has been used to measure the skiers’ position and velocity during cross-country skiing [7,15,16,17]. GNSS/GPS can measure a skier’s position, which would reveal whether he/she skies on the straight section or a corner using a course map. Skiers hardly use a turn in a straight section in skating-style skiing. Hence, V4 in the straight section would be a transition and that in a corner would be a turn, as identified using GNSS/GPS measurements. Nonetheless, some skiers use transitions and turns in succession. Further studies are needed to detect transitions and turns in both laboratory and field conditions.

In this method, the pole contacts were detected, which can be used to calculate the cycle time. Furthermore, pole lift was detected using the norm pole acceleration [13]. The poling time and recovery time can be calculated using pole contact and lift [12,13]. Furthermore, the inertial sensor on the roller ski could detect ski contact and leave. These parameters are useful for spatio-temporal analysis of each subtechnique. In addition, it should be possible to use sensor data to evaluate left/right side symmetry/asymmetry in each subtechnique.

The accurate identification of subtechniques is a gateway to analyze a skier’s performance in a course condition. The identified subtechniques with GNSS/GPS data could analyze what subtechniques were used at a particular position and inclination of the course, the distribution of the main subtechniques, the skiing velocity in each subtechnique, and the comparison of subtechnique selection in each lap [7]. Furthermore, the subtechnique distributions as a function of the inclination and skiing velocity from the combination of subtechnique detection and GNSS/GPS data has been used to analyze the technical characteristics of skiers [14,15]. This information would assist the evaluation of subtechnique selection and the strong and weak subtechniques of a skier. Therefore, the combination of inertial sensors and GNSS/GPS would be useful for analyzing each skier’s subtechniques.

## 5. Conclusions

An automated identification system using data from four inertial sensors mounted on both wrists and roller skis successfully classified the skating-style subtechniques correctly. However, identification accuracy can be improved by separately identifying transitions and turns. Further analysis of arm and ski movements can provide spatio-temporal analysis and symmetry/asymmetry evaluation of each subtechnique. In addition, the use of GNSS/GPS can provide information regarding the skiing velocity, position, and gradient of the terrain. This information and the detected subtechnique can help in analyzing a skier’s performance in course conditions such as subtechnique selection and evaluation of each subtechnique.

## Figures and Tables

**Table 1:** Sequence of each main subtechnique. Row A comprises the first subtechnique of the sequence, Row B the subtechniques that can follow the first one, and Row C the subtechniques that require a transition after the first one.

A	B	C
V1R	V1R	V1L, V2R, V2L, V2AR, V2AL
V1L	V1L	V1R, V2R, V2L, V2AR, V2AL
V2R	V2L, V2AL	V1R, V1L, V2R, V2AR
V2L	V2R, V2AR	V1R, V1L, V2L, V2AL
V2AR	V2L, V2AR	V1R, V1L, V2L, V2AL
V2AL	V2R, V2AL	V1R, V1L, V2R, V2AR

**Table 2:** Anthropometric and physical performance characteristics of the subjects. The data are shown as “means (standard deviation)”.

	Cross-Country		Nordic Combined
	Male (n = 4)	Female (n = 7)	Male (n = 4)
Age (years)	19.0 (2.0)	20.1 (1.5)	19.5 (1.3)
Height (m)	1.78 (0.02)	1.60 (0.05)	1.72 (0.02)
Weight (kg)	71.2 (2.6)	52.7 (3.4)	64.3 (2.7)
VO_2max_ (mL/min/kg)	69.9 (1.7)	57.3 (6.1)	63.3 (3.7)

**Table 3:** Confusion matrix for subtechniques using automatic identification *versus* a visual check.

		Automatic Identification								
		V1R	V1L	V2R	V2L	V2AR	V2AL	V4	Total	Accuracy (%)
Visual check	V1R	**120**	1	1	0	3	0	2	127	94.5
	V1L	0	**40**	0	0	0	0	1	41	97.6
	V2R	0	0	**1920**	1	0	7	20	1950	98.5
	V2L	0	0	0	**1932**	3	0	20	1955	98.8
	V2AR	0	0	7	0	**767**	0	31	805	95.3
	V2AL	0	0	3	15	1	**587**	21	631	93.0
	V4	17	5	39	27	59	44	**1047**	1259	83.2
	Total	137	46	1970	1975	833	638	1142		
	Precision (%)	87.6	87.0	97.5	97.8	92.1	92.0	91.7		
